# Filling the Gap 115 Years after Ronald Ross: The Distribution of the *Anopheles coluzzii* and *Anopheles gambiae* s.s from Freetown and Monrovia, West Africa

**DOI:** 10.1371/journal.pone.0064939

**Published:** 2013-05-31

**Authors:** Dziedzom K. de Souza, Benjamin G. Koudou, Fatorma K. Bolay, Daniel A. Boakye, Moses J. Bockarie

**Affiliations:** 1 Parasitology Department, Noguchi Memorial Institute for Medical Research, University of Ghana, Accra, Ghana; 2 Centre for Neglected Tropical Diseases, Liverpool School of Tropical Medicine, Liverpool, United Kingdom; 3 Liberian Institute for Biomedical Research, Charlesville, Liberia; 4 Centre Suisse de Recherches Scientifiques en Cote d’Ivoire, Abidjan, Cote d’Ivoire; Université Pierre et Marie Curie, France

## Abstract

It was in Freetown, Sierra Leone, that the malaria mosquito *Anopheles coastalis*, now known as *Anopheles gambiae*, was first discovered as the vector of malaria, in 1899. That discovery led to a pioneering vector research in Sierra Leone and neighbouring Liberia, where mosquito species were extensively characterized. Unfortunately, the decade long civil conflicts of the 1990s, in both countries, resulted in a stagnation of the once vibrant research on disease vectors. This paper attempts to fill in some of the gaps on what is now known of the distribution of the sibling species of the *An. gambiae* complex, and especially the *An. coluzzii* and *An. gambiae* s.s, formerly known as the *An. gambiae* molecular M and S forms respectively, in the cities of Freetown and Monrovia.

## Introduction

*Anopheles gambiae* (formally known *An. coastalis*) was first incriminated as a vector of malaria by Sir Ronald Ross in Freetown, Sierra Leone in 1899 [1]. Mosquitoes belonging to the *Anopheles gambiae* complex are the major vectors of malaria in West Africa. As such studying their distribution, ecology and population structure is essential for effective malaria control programs. In the majority of West African countries, various studies on these important vectors have been undertaken in details. However, there is a gap in knowledge of these vectors in post conflict countries such as Sierra Leone and Liberia where ecological studies could not be undertaken in the 1990s and early 2000s because of the civil war that engulfed both countries for more than a decade.

Prior to the civil unrest in these countries, the mosquito species and their distribution were largely documented. Sierra Leone primarily served as the centre of malaria vector research and control, being the place where human malarial parasites were first observed in wild-caught *An. gambiae* and *An. funestus*, in 1899 [1]. By the 1950s extensive studies on human biting mosquitoes in both Sierra Leone [1] and Liberia [2]–[5] had been undertaken. However, these studies were focussed on the morphological identification of these important vectors.

By the 1980s the study of *An. gambiae* vectors of malaria in West Africa had evolved from morphometric to cytotaxonomic studies [6], [7]. The development of the PCR in 1990 [8] led to a further evolution in the identification of the sibling species of the *An. gambiae* complex [9], the M and S molecular forms of the *An. gambiae* s.s. [10] and knockdown resistance markers to insecticides [11], [12] using molecular methods. Following molecular and bionomical evidence, the *An. gambiae* molecular M form has subsequently been renamed *An. coluzzii*, whiles the *An. gambiae* molecular S form retained the name *An. gambiae* s.s [13]. These studies have been undertaken and enhanced malaria control activities in most countries in West Africa. Unfortunately, the civil conflict in Liberia (1989–1996, 1999–2003) and Sierra Leone (1991–2002) stagnated the once vibrant malaria vector research activities in the cities of Monrovia and Freetown. To date, no molecular identification of members of the *An. gambiae* complex had been undertaken/published in Sierra Leone, and in Liberia only one study [14] has described the distribution of the M and S forms of the *An. gambiae* s.s and their *kdr* frequencies in the Bomi County. In this short communication, we report the distribution of the *An. coluzzii* and the *An. gambiae* s.s., in Freetown and Monrovia the capital cities of Sierra Leone and Liberia respectively.

## Methods

### Study Sites

**Figure 1:**
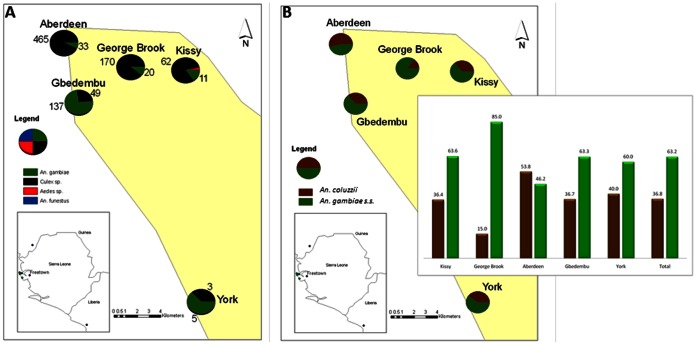
Distribution of the mosquito species, the *An. coluzzii* and the *An. gambiae* s.s from the sample communities in Freetown, Sierra Leone.

**Figure 2:**
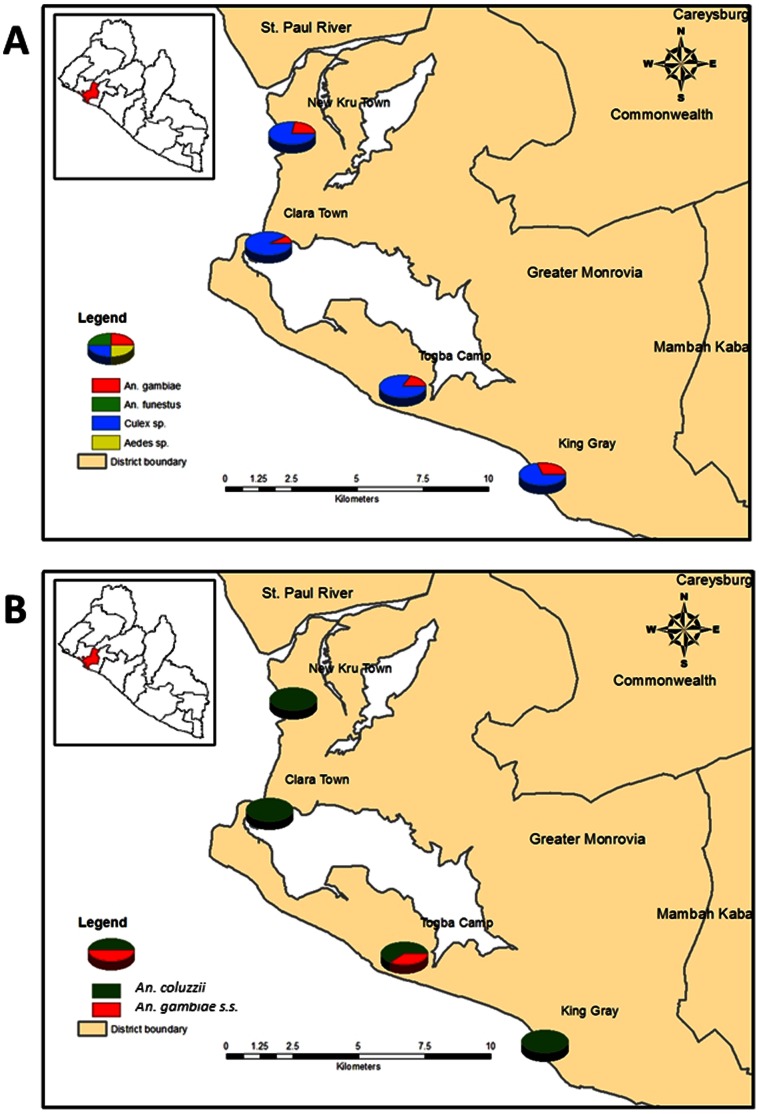
Distribution of the mosquito species, the *An. coluzzii* and the *An. gambiae* s.s from the sample communities in Monrovia, Liberia.

In Sierra Leone, samples were collected in November-December 2009, from 89 households in 5 communities around Freetown. In Liberia, samples were collected in July 2011, from 100 households in 4 communities in Monrovia (See Figures 1 and 2 below). Samples were collected using the pyrethrum spray method. In each community, samples were collected early in the morning between 5 and 9 am, in different households, over a 3 days period.

The collected samples were identified morphologically and the *Anopheles* species were stored individually on silica gel, while the *Culex spp*. specimens were put together in pools of 20. Other mosquito species were also stored separately. The collected samples were brought to the Noguchi Memorial Institute for Medical Research, Ghana, for molecular identification.

### Molecular Studies

Genomic DNA was extracted from 2–3 legs from each mosquito, using the boil preparation method. Briefly, the legs were crushed in 100 µl of distilled water and boiled at 95°C for 10 minutes. The supernatant was used as template for the PCR. The extracted DNA was used for species identification using Polymerase Chain Reaction, Restriction Fragment Length Polymorphism (PCR-RFLP) [10]. The determination of the knockdown mutation conferring resistance to pyrethroids was undertaken using the method of Martinez-Torres et al [11].

### Ethics Statement

Approval for this study was obtained from the IRB of the Liverpool School of Tropical Medicine and the Ethics and Scientific Review Committees of the Ministries of Health in Sierra Leone and Liberia. The communities, where sampling was done, were informed on the project and consent sought from the local authorities within each community. Consent was also sought from the households where mosquito sampling was carried out.

## Results and Discussion

### Specimen Collection and Morphological Identification

The number of collections from each community in Freetown is shown in Table 1. Overall, 206 *An. gambiae* and 749 *Culex* species were collected. In Monrovia, 161 and 381 *An. gambiae* and *Culex* species were collected respectively. *Culex* was the most common mosquito species collected, due to the polluted breeding waters found in the areas where the collections were made. *An. funestus* and *Aedes spp*. were also collected, but in very low numbers. The distribution of the mosquito species collected in Freetown and Monrovia is shown in Figure 1A and 2A respectively.

**Table 1:** Number of mosquito collections from each community in Freetown, Sierra Leone.

Country	Community	*An. gambiae*	*Culex sp.*	*Aedes sp.*	*An. funestus*
**Sierra Leone**	**Kissy**	11	62	2	–
	**George Brook**	20	170	–	–
	**Aberdeen**	33	465	–	–
	**Gbedembu**	137	49	–	3
	**York**	5	3	–	–
	**Total**	**206**	**749**	**2**	**3**
**Liberia**	**Clara Town**	25	70	3	–
	**New Kru Town**	43	72	–	–
	**King Gray**	61	89	–	–
	**Togba Camp**	32	87	–	1
	**Total**	**161**	**318**	**3**	**1**

### Molecular Identification and Distribution of the *An. coluzzii* and *An. gambiae* s.s

194 samples were analyzed for the molecular identification of the members of the *An. gambiae* complex in Freetown. 182 (93.8%) were successfully identified as either *An. coluzzii* or *An. gambiae* s.s. The remaining 12 samples failed to amplify. Further differentiation revealed 63.2% of the samples analyzed to be *An. gambiae* s.s., while the remaining 36.8% were *An. coluzzii*. The distribution of *An. gambiae* s.s. and *An. coluzzii* is shown in Figure 1B below. This study is the first to analyze the distribution of the *An. gambiae* s.s. and *An. coluzzii* in Freetown, Sierra Leone. Sierra Leone is a mountainous, forested region and this may explain the high proportion of *An. gambiae* s.s. compared to *An. coluzzii*, and is in accordance with a study [15] that showed that the *An. gambiae* molecular S form was dominant in mountainous, forested areas.

In Monrovia, 105 mosquitoes morphologically identified as *An. gambiae* were analyzed. Out of these, 90.5% were identified as *An. coluzzii*, and the remaining 9.5% as *An. gambiae* s.s. These results are consistent with the results of Temu and colleagues [14] which showed 98% of samples identified to be the *An. gambiae* molecular M form in the Bomi County. Figure 2B shows the distribution of *An. gambiae* s.s. and *An. coluzzii* in the collection sites in Monrovia.

### Distribution of Knockdown Resistance Mutations

Very high proportions of *kdr* mutations (96.2%) were detected in the *An. gambiae* populations (Table 2). The mutation was present both in *An. gambiae* s.s. and *An. coluzzii*, but was more common in the *An. gambiae* s.s. Few hybrids were also detected. The presence of the *kdr* mutation was initially thought to be only present in the *An. gambiae* molecular S form. However, studies have shown its occurrence in the *An. gambiae* molecular M form [16]–[20]. This study showed the mutation in 91% of the *An. coluzzii* identified, similar to values recently reported in the *An. gambiae* molecular M form in Cote d’Ivoire [20]. It is suggested that the presence of the mutation in the M form may be a result of introgression from the S form [17]. However, observations from the Island of Bioko-Equatorial Guinea, where the mutation was only observed in the M form [19], suggested that the mutation in the M form may have arisen independently due to intensive insecticide application.

**Table 2:** The distribution of the *kdr* allele in the *An. coluzzii* and the *An. gambiae* s.s in Freetown.

		*kdr* Allele		
Communities	*An. spp*	RR	SS	RS
**Aberdeen**	*An. coluzzii*	85.7% (12)	14.3% (2)	–
	*An. gambiae* s.s	100% (12)	–	–
**Gbedembu**	*An. coluzzii*	93.2% (41)	6.8% (3)	–
	*An. gambiae* s.s	97.4% (74)	–	2.6% (2)
**George Brook**	*An. coluzzii*	66.7% (2)	33.3% (1)	
	*An. gambiae* s.s	88.2% (15)	5.9% (1)	5.9% (1)
**Kissi**	*An. coluzzii*	100% (4)	–	–
	*An. gambiae* s.s	100% (7)	–	–
**York**	*An. coluzzii*	100% (2)	–	–
	*An. gambiae* s.s	100% (3)	–	–
**Total**	***An. coluzzii***	**91.1% (61)**	**8.9% (6)**	**–**
	***An. gambiae*** ** s.s**	**96.5% (111)**	**0.9% (1)**	**2.6% (3)**

The very high levels of *kdr* resistance to pyrethroids might be due to the high usage of insecticides (e.g. BHC and DDT) for malaria control activities in Sierra Leone, with possible cross resistance to pyrethroids. Studies conducted in the late 1950’s, on the development of insecticide resistance in the *An. gambiae* in Freetown, showed the factor conferring resistance to BHC in 66% of the population [21]. Dieldrin resistance was observed in 91–100% of *An. gambiae* populations in neighboring Liberia in 1957 [22] and was reported in Freetown in 1966 [23]. HCH and DDT were widely used for house spraying and larviciding in Freetown after the World War II [1]. Temephos and malathion were also used for larviciding until it was realized that control activities were unsuccessful, with a possible resistance to malathion [1]. Despite these reports we cannot conclusively assign the observed high levels of *kdr* only to insecticide use in the 1950’s and further studies will be required to assess this.

While the frequencies of *kdr* mutations were not determined for the Monrovia samples, it is believed that the situation may not be much different from Freetown, considering the mosquito control activities that were in place in the 1950s [1], [24]. Also, recent studies in the Bomi County revealed *kdr* frequencies of over 90% [14]. Although these results may represent initial information, it was recently demonstrated that there is no significant association between the presence of the 1014F *kdr* allele and ability to survive exposure to pyrethroid [20]. Thus insecticide susceptibility testing and bio-assay data are necessary to validate our findings.

### Conclusions

The distribution of the *An. coluzzii* and *An. gambiae* s.s and prevalence of the *kdr* gene are described here for the first time in Sierra Leone, nearly 115 years after the incrimination of the Anopheles mosquito as the vector of malaria. This study showed that the *An. gambiae* s.s is the most dominant sibling species of *An. gambiae* complex in Freetown, while the *An. coluzzii* is the dominant species in Monrovia. A high prevalence of *kdr* mutation has been observed in both *An. coluzzii* and *An. gambiae* s.s in Freetown. There is however a limitation to these results, in that the determination of the *kdr* frequencies was not undertaken on surviving or dead mosquitoes exposed to pyrethroids through insecticide susceptibility testing. However, due to the high levels of *kdr* mutation in the *An. coluzzii* and *An. gambiae* s.s populations, it is recommended that insecticide susceptibility testing be done on non-pyrethroids insecticides (organophosphates and carbamates) in order to determine the insecticide of choice for future vector control activities.
