# Correction: Filling the Gap 115 Years after Ronald Ross: The Distribution of the *Anopheles coluzzii* and *Anopheles gambiae* s.s from Freetown and Monrovia, West Africa

**DOI:** 10.1371/annotation/46a18212-657a-4021-8368-4819fc520cbc

**Published:** 2014-01-02

**Authors:** Dziedzom K. de Souza, Benjamin G. Koudou, Fatorma K. Bolay, Daniel A. Boakye, Moses J. Bockarie

There is an error in the title of Table 1. The corrected title is: Number of mosquito collections from each community in Freetown and Monrovia 

